# Heterogeneous Visual Function Deficits in Intermediate Age-Related Macular Degeneration: A MACUSTAR Report

**DOI:** 10.1016/j.xops.2025.100708

**Published:** 2025-01-13

**Authors:** Hannah M.P. Dunbar, David P. Crabb, Charlotte Behning, Alison M. Binns, Amina Abdirahman, Jan H. Terheyden, Stephen Poor, Robert P. Finger, Sergio Leal, Adnan Tufail, Frank G. Holz, Matthias Schmid, Ulrich F.O. Luhmann, MACUSTAR Consortium

**Affiliations:** 1 Institute of Ophthalmology, London, UK; 2 Moorfields Eye Hospital NHS Foundation Trust, London, UK; 3 City University of London, London, UK; 4 Medical Faculty, Institute of Medical Biometry, Informatics and Epidemiology, University of Bonn, Bonn, Germany; 5 Department of Ophthalmology, University Hospital Bonn, Bonn, Germany; 6 Novartis Pharma, Cambridge; 7 Department of Ophthalmology, Mannheim University Hospital, Heidelberg University, Mannheim, Germany; 8 Bayer Consumer Care AG, Basel, Switzerland; 9 Roche Pharmaceutical Research and Early Development, Translational Medicine Ophthalmology, Roche Innovation Center Basel, Basel, Switzerland

**Keywords:** Age-related macular degeneration, Visual function, Visual dysfunction

## Abstract

**Objective:**

To examine the extent to which visual function in Beckman age-related macular degeneration (AMD) disease stages differs from age-similar peers with no AMD and, using reference limits derived from those with no AMD, test the hypothesis that people with intermediate AMD (iAMD) have heterogeneous visual function deficits.

**Design:**

Cross-sectional analyses of a range of baseline visual function measures from the MACUSTAR study—an international, multicenter (n = 20), noninterventional clinical trial.

**Participants:**

Five hundred eighty-five participants with iAMD (67% female, mean [standard deviation] age 72 [7] years) were recruited alongside 56 with no AMD (59% female, 68 [6]), 34 with early AMD (79% female, 72 [6]), and 43 with late AMD (49% female, 75 [6]).

**Methods:**

Participants performed best-corrected visual acuity (BCVA), low luminance visual acuity (LLVA), Moorfields acuity test (MAT), Pelli-Robson contrast sensitivity (PR-CS), small print standardized International reading speed test (SPS), mesopic and scotopic average threshold (MesAT and ScoAT; macular integrity assessment, iCare), and rod intercept time (RIT; AdaptDx, Lumithera).

**Main Outcome Measures:**

Relationship between each visual function measure and disease classification was examined by linear regression adjusted for age, sex, and phakic status. No AMD data were used to estimate normal reference limits for each visual function test. Intermediate AMD scores were dichotomized against reference limits, and the proportion worse than each limit was calculated.

**Results:**

Relative to no AMD, SPS was significantly worse in early AMD (*P* = 0.001); all measures except SPS were significantly reduced in iAMD (*P* < 0.02), and all measures were markedly reduced in late AMD (*P* < 0.0001). Thirty-one point three percent of iAMD participants breached reference limits for PR-CS, 29.4% for RIT, 24.1% for LLVA, 23.2% for MAT, 20.5% for BCVA, 19.8% for MesAT, 17.9% for ScoAT, and 12.6% for SPS. Of the iAMD participants, 69.6% and 42.7% breached ≥1 and ≥2 reference limits, respectively, whereas 33.6% and 5.7% would be expected by chance.

**Conclusions:**

A large proportion of people with structurally defined iAMD exhibit heterogeneous visual function deficits outside normal reference limits. This observation may be relevant for the design and inclusion criteria of future interventional trials.

**Financial Disclosure(s):**

Proprietary or commercial disclosure may be found in the Footnotes and Disclosures at the end of this article.

Age-related macular degeneration (AMD) is a major cause of severe sight impairment globally affecting 196 million people, projected to rise to 288 million by 2040.^1^ The progressive stages of AMD, referred to as early, intermediate, and late disease, are identified based on the structural features present in color fundus photography.^2^ The value of incorporating OCT features within future classification paradigms is being explored.^3^, ^4^, ^5^ Despite relevance to patients, visual function measures are not currently considered within AMD classification systems and could potentially distinguish structurally similar disease with differing functional impacts, underlying pathology, or responsiveness to therapeutics.

Patient-reported outcome studies suggest that people with intermediate AMD (iAMD) experience difficulty under low luminance conditions.^6^^,^^7^ Multiple measures of visual function under photopic, mesopic, and scotopic conditions are also significantly worse in iAMD compared to healthy controls^8^, ^9^, ^10^, ^11^, ^12^, ^13^, ^14^, ^15^; however, as absolute differences are small, clinical significance is unclear. Substantial functional heterogeneity within measures of low-luminance vision, contrast sensitivity, retinal sensitivity, and rod adaptation have been observed in iAMD,^10^^,^^12^^,^^16^ suggesting that comparing mean visual function measures between disease classifications may miss the presence of subgroups of people with iAMD experiencing meaningful functional impairment. Establishing evidence of visual function heterogeneity in people with iAMD, its prevalence, and the extent to which different dimensions of visual function are affected could be useful for future trial design, regulatory purposes, and studies of new therapies.

Here, we interrogate data from a large multicenter study on a range of clinical visual function assessments to examine the extent to which visual function in AMD stages differs from age-similar peers with no AMD, using reference limits derived from those with no AMD, and test the hypothesis that people with iAMD have heterogeneous visual deficits.

## Methods

MACUSTAR (Registration NCT03349801; www.clinicaltrials.gov) is a noninterventional 20-center clinical trial, the protocol of which has been published previously.^17^ Briefly, MACUSTAR has 2 parts: a cross-sectional study where structural and functional candidate endpoints have been evaluated with respect to their repeatability and ability to distinguish normal aging changes from Beckman-classified^2^ AMD stages (no AMD, early AMD, iAMD, and late AMD [includes both geographic atrophy and neovascular AMD])^18^^,^^19^ and a longitudinal study where the ability of candidate endpoints to detect change over time and predict progression of iAMD to late AMD is being evaluated over a 3-year time course in a larger cohort with iAMD, with an extension to the 6-year follow-up period recently announced. The present work uses the full baseline dataset across both components of the MACUSTAR study.

Written informed consent was obtained from all participants. The research was approved by individual local ethics committees (summarized in^20^) and conformed to the Declaration of Helsinki. Inclusion and exclusion criteria have previously been published.^17^^,^^21^ Disease classification was confirmed by a central reading center based on multimodal imaging (color fundus photography, near-infrared reflectance scanning laser ophthalmoscopy, fundus autofluorescence, and spectral-domain OCT) graded according to a standardized, predefined grading protocol based on the Beckman AMD classification.^2^^,^^22^

All participants performed a battery of visual function assessments including best-corrected visual acuity (BCVA), low luminance visual acuity (LLVA),^23^ Moorfields acuity test (MAT),^24^ Pelli-Robson contrast sensitivity (PR-CS),^25^ small print standardized International reading speed test (SPS),^26^^,^^27^ average threshold from mesopic and scotopic fundus-controlled perimetry (MesAT and ScoAT; macular integrity assessment, iCare), and rod intercept time (RIT) from dark adaptometry (AdaptDx, Lumithera). A full description of all examination procedures including their standardized operating procedures has been published elsewhere.^18^^,^^19^ As MACUSTAR was conceived to examine the potential of candidate endpoints within iAMD, tests were selected with respect to relevance in iAMD, adequate measurement quality, compatibility with repeated standardized administration under multicenter clinical trial conditions, and acceptance by patients and examiners.^17^ All tests were performed monocularly with the study eye (defined as that with better BCVA or that selected by the investigator if BCVA was equal in both eyes). Visual function data were subject to 6 monthly quality control procedures. Mesopic average threshold, ScoAT, and RIT data were assessed for quality and reliability as per their standardized operating procedures so that only high-quality data were retained for analysis. Rod intercept time values were capped at the maximum test duration (30 minutes). The relationship between each visual function measure and Beckman disease classification was plotted and examined by linear regression adjusted for age, sex, and phakic status with Benjamini-Hochberg adjustment for multiple comparisons.

Cross-sectional data from those with no AMD were used to define a reference limit for normal function on each visual function test against which iAMD results were dichotomized. For visual function measures where higher values equate to better function, the reference limit was defined as the fifth percentile of the baseline no-AMD data. For measures where lower values equate to better function, the 95th percentile was used. Percentiles were computed using the default quantile type of the *quantile* function, which corresponds to a continuous sample of the quantile type 7 described here.^28^ The proportion of participants with iAMD exhibiting function worse than each reference limit was calculated, together with the proportion falling outside, or breaching, 0, 1, 2, 3, 4, 5, 6, 7, or 8 reference limits. Missing data points were classified as not exceeding the threshold. An UpSet plot^29^^,^^30^ was used to graphically display the number and variety of reference limits breached. A negative binomial regression model was fitted to investigate the association between the number of breached visual function limits and phakic status. All analyses were performed in R, version 4.3.0 (R Foundation for Statistical Computing).^31^ STrengthening the Reporting of OBservational studies in Epidemiology (STROBE) reporting guidelines were followed.^32^

## Results

**Figure 1:**
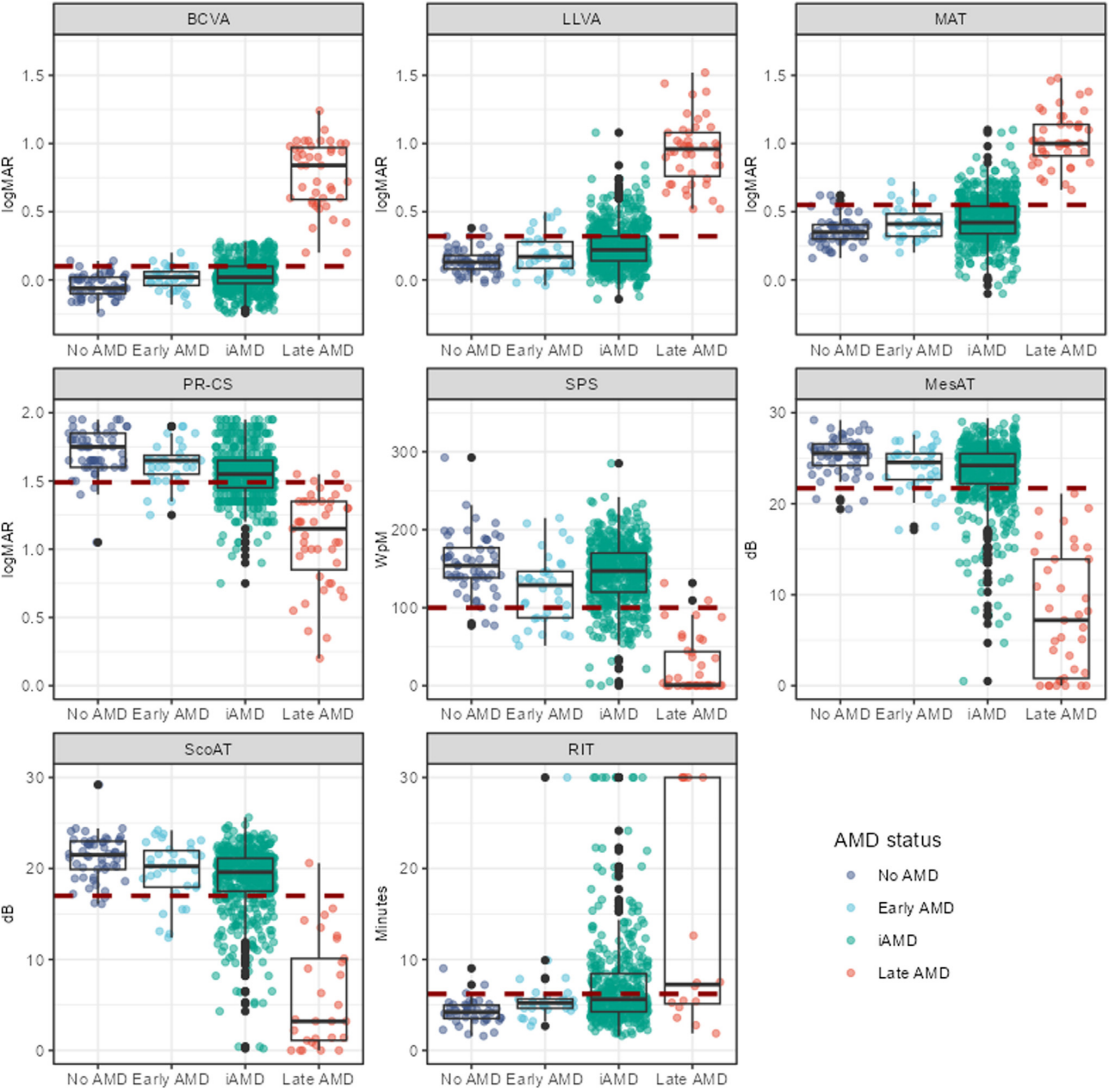
Distribution of the visual function measures by disease classification. Red dashed line indicates reference limit for each test based on no AMD data. AMD = age-related macular degeneration; BCVA = best-corrected visual acuity; dB = decibel; iAMD = intermediate age-related macular degeneration; LLVA = low luminance visual acuity; LogCS = logarithm of contrast sensitivity; logMAR = logarithm of the minimum angle of resolution; MAT = Moorfields acuity test; MesAT = mesopic average threshold; PR-CS = Pelli-Robson contrast sensitivity; RIT = rod intercept time; ScoAT = scotopic average threshold; SPS = small print standardized International reading speed test; wpm = words per minute.

Five hundred and eighty-five participants with iAMD (67% female, mean [± standard deviation] age 72 ± 7 years) were recruited alongside 56 with no AMD (59% female, 68 ± 6 years), 34 with early AMD (79% female, 72 ± 6 years), and 43 with late AMD (49% female, 75 ± 6 years). More than 99% of participants completed BCVA, LLVA, MAT, and PR-CS measures, with 93.7% performing the SPS. Small print standardized International reading speed test was not performed at 1 site (n = 30) where a native language (Danish) test was not available. The proportion of participants able to return a valid MesAT, ScoAT, and RIT measurements was 90.8%, 85.2%, and 69.1%, respectively. Table 1 provides the distribution of demographic and visual function measures by disease classification, presented graphically in Figure 1.

**Table 1:** Summary of Demographic and Visual Function Measures

	No AMD (n = 56)	Early AMD (n = 34)	Intermediate AMD (n = 585)	Late AMD (n = 43)
Age, yrs				
Mean (SD)	68 (6)	72 (6)	72 (7)	75 (6)
Median [Min, Max]	68 [55, 88]	72 [57, 82]	72 [55, 88]	75 [64, 84]
Sex				
Female	33 (58.9%)	27 (79.4%)	389 (67%)	21 (48.8%)
Male	23 (41.1%)	7 (20.6%)	196 (33%)	22 (51.2%)
Best-corrected visual acuity (BCVA), logMAR				
Mean (SD)	−0.04 (∼20/20) (0.08)	0.01 (∼20/20) (0.08)	0.03 (∼20/20) (0.10)	0.77 (∼20/125) (0.25)
Median [Min, Max]	−0.06 (∼20/16) [−0.24,0.14]	0.02 (∼20/20) [−0.18, 0.20]	0.02 (∼20/20) [−0.24, 0.28]	0.84 (∼20/125) [0.20,1.24]
Missing	0	0	1 (0.2%)	0
Low luminance visual acuity (LLVA), logMAR				
Mean (SD)	0.14 (∼20/25) (0.09)	0.19 (∼20/32) (0.14)	0.24 (∼20/32) (0.16)	0.95 (∼20/200) (0.24)
Median [Min, Max]	0.13 (∼20/25) [−0.02, 0.38]	0.17 (∼20/32) [−0.04, 0.50]	0.22 (∼20/32) [−0.14, 1.08]	0.96 (∼20/200) [0.52, 1.52]
Missing	0	0	2 (0.3%)	0
Moorfields acuity test (MAT), logMAR				
Mean (SD)	0.36 (∼20/50) (0.11)	0.42 (∼20/50) (0.12)	0.44 (∼20/50) (0.16)	1.03 (∼20/200) (0.20)
Median [Min, Max]	0.35 (∼20/50) [0.16, 0.62]	0.41 (∼20/50) [0.20, 0.72]	0.42 (∼20/50) [−0.10, 1.10]	1.00 (∼20/200) [0.66, 1.48]
Missing	0	0	1 (0.2%)	0
Pelli-Robson contrast sensitivity (PR-CS), LogCS				
Mean (SD)	1.71 (0.16)	1.63 (0.16)	1.55 (0.18)	1.07 (0.34)
Median [Min, Max]	1.75 [1.05, 1.95]	1.65 [1.25, 1.90]	1.55 [0.75, 1.95]	1.15 [0.20, 1.55]
Missing	0	0	2 (0.3%)	0
Small print standardized International reading speed test (SPS), wpm				
Mean (SD)	156 (38)	123 (44)	144 (40)	25 (36)
Median [Min, Max]	154 [77, 293]	129 [51, 215]	147 [0, 285]	1 [0, 132]
Missing∗	1 (1.8%)	0 (0%)	37 (6.3%)	4 (9.3%)
Mesopic average threshold (MesAT), dB				
Mean (SD)	25.4 (2.06)	23.9 (2.61)	23.3 (3.65)	7.92 (6.85)
Median [Min, Max]	25.6 [19.4, 29.2]	24.6 [17.1, 27.6]	24.2 [0.50, 29.4]	7.20 [0, 21.1]
Missing	2 (3.6%)	0 (0%)	58 (9.9%)	6 (14.0%)
Scotopic average threshold (ScoAT), dB				
Mean (SD)	21.30 (2.44)	19.60 (3.27)	18.70 (3.78)	6.0 (6.0)
Median [Min, Max]	21.5 [16.1, 29.2]	20.3 [12.4, 24.2]	19.6 [0.20, 25.6]	3.20 [0, 20.6]
Missing	3 (5.4%)	0 (0%)	89 (15.2%)	14 (32.6%)
Rod intercept time, (RIT) at 12° inferiorly, min				
Mean (SD)	4.24 (1.36)	6.15 (4.81)	7.21 (5.07)	13.4 (11.8)
Median [Min, Max]	4.20 [1.58, 9.02]	5.21 [2.68, 30.0]	5.62 [1.59, 30.0]	7.25 [1.87, 30.0]
Missing	13 [23.2%]	5 (14.7%)	177 (30.3%)	27 (62.8%)

∼20/XX = approximate Snellen equivalent; AMD = age-related macular degeneration; dB = decibels; LogCS = logarithm of contrast sensitivity; logMAR = logarithm of the minimum angle of resolution; Max = maximum; Min = minimum; SD = standard deviation; wpm = words per minute.

Summary of demographic and visual function measures segregated by Beckman disease classification.

∗ Thirty participants without access to Danish language IReST included in missing data rate.

A linear regression model adjusted for age, sex, and phakic status examined the relationship between each visual function measure and disease classification, where no AMD was the reference level. Model results are summarized in Table 2. Relative to no AMD, only SPS was significantly worse on average in early AMD (*P* = 0.001), whereas all measures apart from SPS were significantly worse in iAMD (*P* < 0.02). Though statistically significant, in each case model estimates were smaller than the limits of agreement defined during the cross-sectional part of MACUSTAR.^18^^,^^19^ All visual function measures were significantly and markedly poorer in late AMD relative to no AMD (*P* < 0.0001), with all estimates being between 1.6 and 5 times larger than the limits of agreement defined on the MACUSTAR late AMD cohort.^18^^,^^19^ Additionally, age was associated with all visual function measures except for RIT (*P* < 0.0003).

**Table 2:** Relationship between Visual Function Measures and Disease Classification

Visual Function Measure	No AMD vs. Early AMD		No AMD vs. iAMD		No AMD vs. Late AMD	
	Estimate (CI)	Adjusted *P* Value	Estimate (CI)	Adjusted *P* Value	Estimate (CI)	Adjusted *P* Value
BCVA (logMAR)	0.04 (−0.10, 0.08)	0.22	**0.05 (0.02, 0.09)**	**0.0017**	**0.79 (0.74, 0.83)**	**<0.0001**
	No AMD n = 56 Early AMD n = 34		No AMD n = 56 iAMD n = 584		No AMD n = 56 Late AMD n = 43	
LLVA (logMAR)	0.03 (−0.03, 0.09)	0.47	**0.08 (0.04, 0.12)**	**0.0004**	**0.77 (0.71, 0.83)**	**<0.0001**
	No AMD n = 56 Early AMD n = 34		No AMD n = 56 iAMD n = 583		No AMD n = 56 Late AMD n = 43	
MAT (logMAR)	0.03 (−0.03, 0.09)	0.47	**0.06 (0.01, 0.10)**	**0.02**	**0.63 (0.57, 0.69)**	**<0.0001**
	No AMD n = 56 Early AMD n = 34		No AMD n = 56 iAMD n = 584		No AMD n = 56 Late AMD n = 43	
PR-CS (LogCS)	−0.06 (−0.14, 0.02)	0.21	**−0.14 (−0.19, −0.08)**	**<0.0001**	**−0.59 (−0.67, −0.52)**	**<0.0001**
	No AMD n = 56 Early AMD n = 34		No AMD n = 56 iAMD n = 583		No AMD n = 56 Late AMD n = 43	
SPS (wpm)	**−31 (−48, −14)**	**0.001**	−10 (−21, 2)	0.17	**−125 (−141, −109)**	**<0.0001**
	No AMD n = 55 Early AMD n = 34		No AMD n = 55 iAMD n = 548		No AMD n = 55 Late AMD n = 39	
MesAT (dB)	−1.13 (−2.72, 0.46)	0.27	**−1.69 (−2.73, −0.65)**	**0.004**	**−16.61 (−18.17, −15.05)**	**<0.0001**
	No AMD n = 54 Early AMD n = 34		No AMD n = 54 iAMD n = 527		No AMD n = 54 Late AMD n = 37	
ScoAT (dB)	−1.41 (−3.04, 0.22)	0.17	**−2.29 (−3.37, −1.21)**	**0.0001**	**−14.56 (−16.27, −12.84)**	**<0.0001**
	No AMD n = 53 Early AMD n = 34		No AMD n = 53 iAMD n = 496		No AMD n = 53 Late AMD n = 29	
RIT (min)	1.41 (−1.00, 3.82)	0.37	**2.35 (0.72, 3.98)**	**0.01**	**8.32 (5.36, 11.28)**	**<0.0001**
	No AMD n = 43 Early AMD n = 29		No AMD n = 43, iAMD n = 408		No AMD n = 43 Late AMD n = 16	

AMD = age-related macular degeneration; CI = 95% confidence interval; BCVA = best-corrected visual acuity; dB = decibel; iAMD = intermediate age-related macular degeneration; LLVA = low luminance visual acuity; LogCS = logarithm of contrast sensitivity; logMAR = logarithm of the minimum angle of resolution; MAT = Moorfields acuity test; MesAT = Mesopic average threshold; PR-CS = Pelli-Robson contrast sensitivity; RIT = rod intercept time; ScoAT = scotopic average threshold; SPS = small print standardized International reading speed test; wpm = words per minute.

Linear regression model examining the relationship between each visual function measure (as dependent variable) and disease classification, adjusted for age, sex, and phakic status.

Bold indicates significant result.

Calculated reference limits and the proportion of iAMD participants breaching said limits for each visual function test are provided in Table 3 and shown in Figure 1 as a red dashed line. The proportion of those with iAMD breaching individual reference limits was largest for PR-CS (31.3%), followed by RIT (29.4%), LLVA (24.1%), and MAT (23.2%). Roughly one-fifth breached BCVA (20.5%), MesAT (19.8%), and ScoAT (17.9%) reference limits, dropping to an eighth for SPS (12.6%). Average differences between each impaired subgroup and the no AMD group were calculated and are shown in Table 3. The impaired subgroups for BCVA, LLVA, and MAT were between 0.22 logarithm of the minimum angle of resolution (logMAR; 11 letters) and 0.32 LogMAR (16 letters) poorer than the no AMD group. Pelli-Robson contrast sensitivity was 0.35 logarithm of contrast sensitivity (7 letters) poorer, SPS reading speed was 82 words per minute (wpm) slower, MesAT and ScoAT were 7.2 dB and 8.4 dB lower, respectively, and RIT was 7.89 minutes slower.

**Table 3:** Summary of iAMD Participants Breaching Visual Function Reference Limits

Visual Function Measure	Reference Limit	n (%) of iAMD Participants Breaching Reference Limit	iAMD (Mean ± SD)		No AMD (Mean ± SD)	Δ |iAMD (Function Impaired – No AMD)
			Function Impaired	Function Not Impaired		
BCVA (logMAR)	>0.10	120 (20.5%)	0.18 (0.05)	−0.01 (0.08)	−0.04 (0.08)	0.22 (11 letters)
LLVA (logMAR)	>0.32	141 (24.1%)	0.46 (0.12)	0.18 (0.09)	0.14 (0.09)	0.32 (16 letters)
MAT (logMAR)	> 0.55	136 (23.2%)	0.65 (0.09)	0.38 (0.12)	0.36 (0.11)	0.29 (14.5 letters)
PR-CS (LogCS)	<1.49	183 (31.3%)	1.36 (0.12)	1.64 (0.12)	1.71 (0.16)	−0.35 (7 letters)
SPS (wpm)	<100	74 (12.6%)	74 (23)	155 (29)	156 (38)	−82
MesAT (dB)	<21.7	116 (19.8%)	18.2 (4.2)	24.8 (1.6)	25.4 (2.1)	−7.2
ScoAT (dB)	<17.0	105 (17.9%)	12.9 (3.7)	20.3 (1.7)	21.3 (2.4)	−8.4
RIT (mins)	>6.21	172 (29.4%)	12.10 (11.6)	4.39 (1.07)	4.24 (1.36)	−7.86

AMD = age-related macular degeneration; BCVA = best-corrected visual acuity; dB = decibel; iAMD = intermediate age-related macular degeneration; LLVA = low luminance visual acuity; LogCS = logarithm of contrast sensitivity; logMAR = logarithm of the minimum angle of resolution; MAT = Moorfields acuity test; MesAT = mesopic average threshold; PR-CS = Pelli-Robson contrast sensitivity; RIT = rod intercept time; SD = standard deviation; ScoAT = scotopic average threshold; SPS = small print standardized International reading speed test; wpm = words per minute.

Number and proportion of iAMD participants breaching the reference limit for each visual function test calculated as a proportion of the complete iAMD cohort (585). Mean ± standard deviation of those breaching the reference limited (functionally impaired) and not breaching the reference limit (function not impaired) for each variable. No AMD data provided for comparison between iAMD function impaired and no AMD.

Four hundred seven (69.6%) iAMD participants breached the no AMD reference limits on ≥1 visual function test, with 250 (42.7%) breaching ≥2. Binomial probability calculations were used to determine how many participants would be expected to exceed ≥1 ([1 − 1∗(1-0.05)ˆ8] = 33.6%) and ≥2 ([1 − 1∗(1-0.05)ˆ8 − 8!/7!∗0.05∗(1-0.05)ˆ7] = 5.7%) limits by chance under the null hypothesis that people exhibiting function worse than the reference limit have equivalent visual function to peers with no AMD. The number and proportion of those with iAMD who breached 0–8 reference limits are provided in Table S4 (available at www.ophthalmologyscience.org).

**Figure 2:**
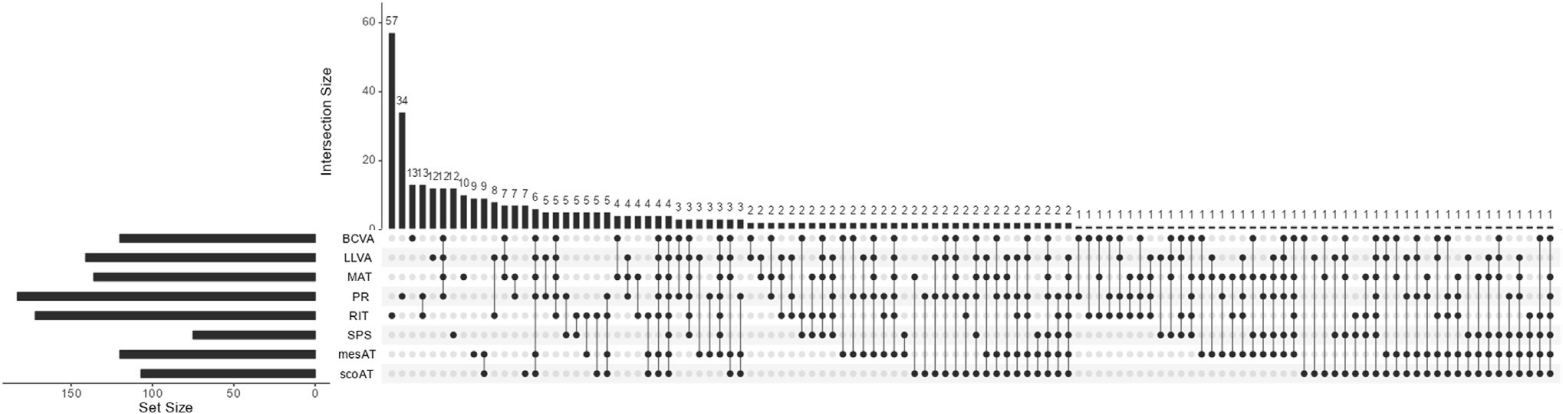
UpSet plot describing the number and extent of reference limits breached in participants with iAMD. Horizontal black bars indicate the set size or number of iAMD participants who breached the reference limit for each visual function (VF) test shown by the adjacent label. Vertical black bars indicate the intersection size or number of iAMD participants who breached the reference limit of the VF test(s) indicated by the filled black circles beneath. For example, the leftmost vertical black bar indicates that 59 iAMD participants breached the RIT reference limit only, while the rightmost vertical black bar indicates that 4 iAMD participants breached the reference limit on all 8 visual function tests. BCVA = best-corrected visual acuity; iAMD = intermediate age-related macular degeneration; LLVA = low luminance visual acuity; MAT = Moorfields acuity test; MesAT = mesopic average threshold; PR-CS = Pelli-Robson contrast sensitivity; RIT = rod intercept time; ScoAT = scotopic average threshold; SPS = small print standardized International reading speed test.

The UpSet plot in Figure 2 graphically displays the number of iAMD participants who breached the reference limit for each visual function test and the extent to which iAMD participants breached reference limits on single or multiple visual function tests. Though the PR-CS reference limit was breached most commonly overall, RIT was the most common reference limit breached in isolation. Individuals who breached the PR-CS limit more often breached ≥1 additional limits in combination. The most common combination of 2 reference limits breached was PR-CS and MAT (n = 134, [22.9%]), with RIT and SPS being the least common (n = 47, [8.0%]). Four individuals exceeded all 8 limits. No association was found between the number of breached visual function limits and phakic status (*P* > 0.16).

Because reference limits calculated for these analyses account for measurement variability between those with no AMD but not within individuals, a sensitivity analysis was performed exploiting no AMD data obtained at both baseline (day 0) and validation (day 14 ± 7) study visits. Here, secondary reference limits for each visual function measure were calculated from a dataset containing the poorer of the 2 measurements recorded at these 2 study visits from no AMD participants. Results are provided in Tables S5 and S6 (available at www.ophthalmologyscience.org). Application of secondary reference limits revealed that 360 (61.5%) iAMD participants breached ≥1 limit and 209 (35.7%) breached ≥2.

## Discussion

In this large, multicenter dataset, a range of visual function tests did not show clinically meaningful average differences in functional performance between normal aging and both early AMD and iAMD. Conversely, visual function in those with late AMD was markedly and significantly reduced, exceeding the limits of agreement defined for the MACUSTAR visual function test battery by between 1.6 and 5 times. Despite average visual function in iAMD being clinically comparable to no AMD on a population level, 69.6% of iAMD participants had deficits in ≥1 visual function test falling outside reference limits established in visually healthy peers—more than twofold greater than that expected by chance. Additionally, 42.7% of participants with iAMD had deficits in ≥2 visual function tests—7 times more than that expected by chance. Estimates of the proportion affected by chance assume that tests are unrelated. Correlation coefficients between the visual function measures in this cohort are in the weak to moderate range.^33^ Taken together, this supports the notion that functional heterogeneity in the baseline iAMD population of MACUSTAR cannot be explained as a chance finding. That said, the observed proportions depend on the veracity of the reference limits used.

There are no universally accepted thresholds for normal function in older eyes. Therefore, we defined reference limits on data from 56 visually healthy peers in the same study. This dataset has the unique advantage of being obtained under the same multicenter, multitechnician conditions, using the same publicly available standardized operating procedures.^18^^,^^19^ We additionally exploited the availability of repeat no AMD visual function data to assess the impact of intraobserver variability on our calculated reference limits. This sensitivity analysis adopted the cautious approach of basing a set of secondary reference limits on the worst of 2 visual function measurements. Comparing these to our initial limits showed that for letter-scored tests (BCVA, LLVA, MAT, and PR-CS), reference limits differed by between 0 and 1.5 letters. Small print standardized International reading speed test limits differed by 3 wpm, microperimetry average threshold measures by between 0.8 and 1 dB, and RIT by 0.27 minutes. Logically, application of these adjusted thresholds resulted in a smaller proportion of iAMD participants outside the reference limits; however, the proportion outside ≥1 (61.5%) and 2 (35.7%) limits was roughly 1.8 times and 6 times that which was expected by chance, respectively, corroborating our primary finding that a large proportion of participants with iAMD have deficits in visual function falling outside the reference limits established in visually healthy peers.

A comparative study of visual function in normal controls and iAMD assessed BCVA, LLVA, MAT, PR-CS, SPS, MesAT, and ScoAT in 24 control eyes in a single center (61.7 ± 6.1 years) using equivalent equipment and testing protocols.^9^ Using their published no AMD data to calculate the mean ± 2 × standard deviation for each visual function measure as a proxy for the fifth/95th percentile revealed roughly equivalent values to our reference limits (BCVA: 0.12 logMAR; LLVA: 0.38 logMAR; MAT: 0.50 logMAR; PR-CS: 1.50 logarithm of contrast sensitivity; SPS: 116 wpm; MesAT: 22.7 dB; ScoAT: 19.5 dB). The single-center ALSTAR2 study has also assessed a range of visual function parameters in 239 people (70.8 ± 5.6 years) in normal macular health (Age-Related Eye Disease Study^34^ grade 1).^13^^,^^35^ Though defining reference limits was not the primary aim of ALSTAR2, as one of the largest published studies of normal macular health, it serves as a very useful comparator. Furthermore, there is some overlap between the visual function test batteries of ALSTAR2 and MACUSTAR (both assess BCVA, LLVA, contrast sensitivity, MesAT, ScoAT, and RIT), though the testing equipment and protocols differ. These factors limit a true, direct comparison. Nevertheless, proxy reference limits calculated using baseline ALSTAR2 control data (using the method described previously) reveal slightly more conservative values than our reference limits for all tests except RIT (BCVA: 0.15 logMAR; LLVA: 0.42 logMAR; MARS contrast sensitivity^36^: 1.39 logarithm of contrast sensitivity; MesAT: 19.1 dB; ScoAT: 16.0 dB). A direct comparison for RIT is more challenging as test parameters differ. Based on data from the same 12° retinal location used in MACUSTAR, though using a higher bleach and longer maximum test duration,^35^ the proxy RIT limit was 16.2 minutes. Recent evidence suggests that dark adaptation deficits in early AMD are likely greatest when assessed at 5° eccentricity.^35^ In MACUSTAR, the 12° test location was chosen based on pilot data showing that a deficit is present in people with iAMD at 12° and that a smaller proportion of participants would demonstrate a ceiling effect within a clinically practical test duration.^37^, ^38^, ^39^ In line with this pilot data, our results support the existence of an RIT deficit at 12°, as a higher proportion of participants fell outside the RIT reference limit than any other functional parameter except for PR-CS. However, we note that a more centrally located target may have identified an even higher proportion of individuals with abnormal RIT, had the test duration been extended to 45 or 60 minutes. In addition to test parameter differences and the different method of reference limit calculation, the slightly older age of the ALSTAR2 cohort ([70.8 ± 5.6] vs. [68 ± 6] years) may also contribute to the difference in reference limits between studies.

MACUSTAR reference limits presented here cannot be considered true normative cutoff values given the small dataset on which they are based; this is a limitation. Nevertheless, we suggest that this method of defining reference limits for exposing functional heterogeneity is justified by its statistical underpinning, consensus with previous work, and cautious nature. However, future work characterizing normative visual function on the MACUSTAR test battery in a larger cohort with a wider and balanced age range is warranted to fully explore the concept of functional heterogeneity in iAMD and other ocular disease cohorts.

Functional heterogeneity in Age-Related Eye Disease Study-defined iAMD has been previously observed based on mesopic microperimetry, low luminance deficit, and dark adaptation measures in single-center studies.^37^^,^^39^^,^^40^ Here, we add further evidence that this heterogeneity extends to a wider range of clinical visual function tests and is observable in a large, multicenter population of people with Beckman-classified iAMD examined under clinical trial conditions. Recent work using qualitative autofluorescence to assess early changes in AMD suggests that some eyes classified as Beckman iAMD may be at an earlier disease stage.^41^ This suggests functional heterogeneity may not only be the preserve of iAMD but may also extend to those with earlier disease.

Though a certain degree of heterogeneity could be introduced by technical variability or execution, especially in a multicenter setting, efforts were employed to minimize this. Technicians were certified, and 6-monthly quality control assessments were performed to recognize any additional training needs and identify and exclude invalid data. Furthermore, test-retest variability was determined for all tests^18^^,^^19^ and pilot testing was performed to optimize test parameters.^38^^,^^42^ Thus, we consider our data to have high quality and that our conclusions are valid.

The average differences between the iAMD subgroup with impaired function and normal peers exceed the test-retest limits for each visual function test,^18^^,^^19^ supporting the clinical relevance of functional heterogeneity in iAMD. Furthermore, the differences approximate the changes proposed to represent clinical relevance (15-letters on acuity tests,^43^ 6-letters on PR-CS,^44^^,^^45^ 80 wpm on SPS,^46^^,^^47^ 7 dB in retinal sensitivity,^48^ and 6.5 minutes on RIT, albeit at a different retinal location^49^) based on methods including expert consensus, association of functional measures with task performance or self-report, and diagnostic sensitivity and specificity.

Deficits were most commonly found in PR-CS and RIT; however, PR-CS deficits occurred more often in combination with other deficits, while RIT deficits were more frequently seen in isolation, suggesting the possibility of distinct functional profiles within the structural classification of iAMD. For example, given that delayed RIT in normal macular health is associated with development of incident AMD after 3 years,^50^ those with RIT deficits may be at an earlier stage of progression than those who have accumulated multiple visual function deficits. It is also accepted that functional performance in iAMD varies with and without reticular pseudodrusen.^35^^,^^51^, ^52^, ^53^, ^54^, ^55^ As such, differing functional outcomes may be associated with distinct structural phenotypes.

Given the functional impact of cataract, we were reassured that phakic status was not related to the number of breached visual function limits. Age, however, was associated with all visual function measures apart from RIT. If age deputizes for disease duration, functional heterogeneity may in part be explained by various stages of progression within the baseline iAMD cohort rather than visual deficits indicating faster progression toward late disease. That said, 539 of 585 (92%) iAMD cohort participants had bilateral iAMD, with the remainder (46 of 585 [8%]) having iAMD in the study eye and late AMD in the fellow eye. With late AMD in the fellow eye associated with higher rates of progression to late disease,^56^ symmetrical disease in the vast majority of the iAMD population may reduce the likelihood that the heterogeneity observed is the result of differing stages of progression. We acknowledge that chronological, not biological, age was adjusted for. It has been shown that those with a higher biological, than chronological age, are at higher risk of poorer health outcomes, which may be influencing the heterogeneity observed.^57^ We will shortly investigate whether iAMD associated with functional deficits increases the risk of progression to late AMD with longitudinal MACUSTAR data. If so, this may go toward supporting the clinical relevance of functional impairment in iAMD and its potential to be a treatment indication in itself.

Functional heterogeneity may also have a substantial bearing on inclusion criteria for future interventional trials. If criteria are based solely on structural classification, it risks the recruitment of a cohort with an assorted or variable profile of visual function deficits. If, as regulators prefer, visual function endpoints are employed, baseline variation within the assessed visual domain may obscure any potential intervention-related signal.

There are further limitations in this work that should be considered. As described above, the calculation of reference limits is based on a limited sample of 56 no AMD participants. Furthermore, the small size of the early (n = 34) and late AMD (n = 43) groups is also a limitation. The rationale for our sample sizes has been explained previously.^18^ That visual function tests were not chosen based on AMD pathogenesis could be considered a limitation; however, this was not customary at the time of study design. Rather, as described in the methods section, clinical data informed test selection with an emphasis on tests that could potentially be adopted in multicenter clinical trial settings.

We conclude that, when multiple domains of visual function in normal aging are compared to early AMD and iAMD at the population level, average differences across groups are not clinically meaningful, being considerably less than the limits of agreement. However, population-level change may obscure person-level functional decline in iAMD. Using reference limits established in visually healthy peers, 69.6% of those with structurally defined iAMD have ≥1 functional deficit—more than twofold that expected by chance; 42.7% have ≥2 deficits—7 times greater than chance. Average differences between those with iAMD who display functional impairment and those with no AMD approximate clinically meaningful change across visual function assessments. This evidence of visual function heterogeneity in iAMD in our large, multicenter cohort may be relevant to the design and participant inclusion criteria of future intervention iAMD trials, especially those aiming to halt or slow photoreceptor degeneration and loss. It remains to be seen whether people with iAMD who have specific visual function deficits are more likely to progress to late AMD or whether these findings are a reflection of various stages of progression within the MACUSTAR iAMD cohort.

## References

[1] Wong W.L., Su X., Li X. (2014). Global prevalence of age-related macular degeneration and disease burden projection for 2020 and 2040: a systematic review and meta-analysis. Lancet Global Health.

[2] Ferris F.L., Wilkinson C.P., Bird A. (2013). Clinical classification of age-related macular degeneration. Ophthalmology.

[3] Guymer R.H., Rosenfeld P.J., Curcio C.A. (2020). Incomplete retinal pigment epithelial and outer retinal atrophy in age-related macular degeneration: classification of atrophy meeting report 4. Ophthalmology.

[4] Jaffe G.J., Chakravarthy U., Freund K.B. (2021). Imaging features associated with progression to geographic atrophy in age-related macular degeneration: classification of atrophy meeting report 5. Ophthalmology Retina.

[5] Wu Z., Pfau M., Blodi B.A. (2022). OCT signs of early atrophy in age-related macular degeneration: interreader agreement: classification of atrophy meetings report 6. Ophthalmology Retina.

[6] McGuinness M.B., Fraser R.G., Tan R. (2020). Relationship between rod-mediated sensitivity, low-luminance visual acuity, and night vision questionnaire in age-related macular degeneration. Trans Vis Sci Technol.

[7] Thompson A.C., Luhmann U.F.O., Stinnett S.S. (2018). Association of low luminance questionnaire with objective functional measures in early and intermediate age-related macular degeneration. Invest Ophthalmol Vis Sci.

[8] Pondorfer S.G., Wintergerst M.W.M., Gorgi Zadeh S. (2020). Association of visual function measures with drusen volume in early stages of age-related macular degeneration. Invest Ophthalmol Vis Sci.

[9] Pondorfer S.G., Heinemann M., Wintergerst M.W.M. (2020). Detecting vision loss in intermediate age-related macular degeneration: a comparison of visual function tests. PLoS One.

[10] Cocce K.J., Stinnett S.S., Luhmann U.F.O. (2018). Visual function metrics in early and intermediate dry age-related macular degeneration for use as clinical trial endpoints. Am J Ophthalmol.

[11] Chandramohan A., Stinnett S.S., Petrowski J.T. (2016). Visual function measures in early and intermediate age-related macular degeneration. Retina.

[12] Wu Z., Ayton L.N., Guymer R.H., Luu C.D. (2014). Low-luminance visual acuity and microperimetry in age-related macular degeneration. Ophthalmology.

[13] Owsley C., Swain T.A., McGwin G. (2022). How vision is impaired from aging to early and intermediate age-related macular degeneration: insights from ALSTAR2 baseline. Trans Vis Sci Technol.

[14] Vujosevic S., Smolek M.K., Lebow K.A. (2011). Detection of macular function changes in early (AREDS 2) and intermediate (AREDS 3) age-related macular degeneration. Ophthalmologica.

[15] Guymer R.H., Tan R.S., Luu C.D. (2021). Comparison of visual function tests in intermediate age-related macular degeneration. Trans Vis Sci Technol.

[16] Csaky K.G. (2023). Cross-sectional study of cone function in age-related macular degeneration subjects with non-foveal nascent geographic atrophy. Am J Ophthalmol.

[17] Finger R.P., Schmitz-Valckenberg S., Schmid M. (2019). MACUSTAR: development and clinical validation of functional, structural, and patient-reported endpoints in intermediate age-related macular degeneration. Ophthalmologica.

[18] Dunbar H.M., Behning C., Abdirahman A. (2022). Repeatability and discriminatory power of chart-based visual function tests in individuals with age-related macular degeneration: a MACUSTAR study report. JAMA Ophthalmol.

[19] Higgins B.E., Montesano G., Dunbar H.M.P. (2023). Test-retest variability and discriminatory power of measurements from microperimetry and dark adaptation assessment in people with intermediate age-related macular degeneration–A MACUSTAR study report. Trans Vis Sci Technol.

[20] Terheyden J.H., Behning C., Lüning A. (2021). Challenges, facilitators and barriers to screening study participants in early disease stages-experience from the MACUSTAR study. BMC Med Res Methodol.

[21] Terheyden J.H., Holz F.G., Schmitz-Valckenberg S. (2020). Clinical study protocol for a low-interventional study in intermediate age-related macular degeneration developing novel clinical endpoints for interventional clinical trials with a regulatory and patient access intention-MACUSTAR. Trials.

[22] Saßmannshausen M. (2022). Intersession repeatability of structural biomarkers in early and intermediate age-related macular degeneration: a MACUSTAR study report. Trans Vis Sci Technol.

[23] Sunness J.S., Rubin G.S., Broman A. (2008). Low luminance visual dysfunction as a predictor of subsequent visual acuity loss from geographic atrophy in age-related macular degeneration. Ophthalmology.

[24] Shah N., Dakin S.C., Dobinson S. (2016). Visual acuity loss in patients with age-related macular degeneration measured using a novel high-pass letter chart. Br J Ophthalmol.

[25] Pelli D.G., Robson J.G., Wilkins A.J. (1988). The design of a new letter chart for measuring contrast sensitivity. Clin Vis Sci.

[26] Hahn G.A., Penka D., Gehrlich C. (2006). New standardised texts for assessing reading performance in four European languages. Br J Ophthalmol.

[27] Trauzettel-Klosinski S., Dietz K., Group I.R.S. (2012). Standardized assessment of reading performance: the new international reading speed texts IReST. Invest Ophthalmol Vis Sci.

[28] Hyndman R.J., Fan Y. (1996). Sample quantiles in statistical packages. Am Statistician.

[29] Lex A., Gehlenborg N., Strobelt H. (2014). UpSet: visualization of intersecting sets. IEEE Trans Visual Comput Graph.

[30] Conway J.R., Lex A., Gehlenborg N. (2017). UpSetR: an R package for the visualization of intersecting sets and their properties. Bioinformatics.

[31] R Developement Core Team (2009). A language and environment for statistical computing. https://www.R-project.org.

[32] Vandenbroucke J.P., von Elm E., Altman D.G. (2007). Strengthening the reporting of observational studies in epidemiology (STROBE): explanation and elaboration. Ann Intern Med.

[33] Terheyden J.H., Holz F.G., Behning C. (2025). The spectrum of functional, structural and patient-reported outcomes in intermediate age-related macular degeneration–a MACUSTAR study report. Ophthalmologica.

[34] Davis M.D., Gangnon R.E., Lee L.Y. (2005). The Age-Related Eye Disease Study severity scale for age-related macular degeneration: AREDS report No. 17. Arch Ophthalmol.

[35] Owsley C., Swain T.A., McGwin G. (2023). Biologically guided optimization of test target location for rod-mediated dark adaptation in age-related macular degeneration: Alabama study on early age-related macular degeneration 2 baseline. Ophthalmol Sci.

[36] Arditi A. (2005). Improving the design of the letter contrast sensitivity test. Invest Ophthalmol Vis Sci.

[37] Owsley C., Clark M.E., McGwin G. (2017). Natural history of rod-mediated dark adaptation over 2 Years in intermediate age-related macular degeneration. Trans Vis Sci Technol.

[38] Binns A.M., Taylor D.J., Edwards L.A., Crabb D.P. (2018). Determining optimal test parameters for assessing dark adaptation in people with intermediate age-related macular degeneration. Invest Ophthalmol Vis Sci.

[39] Nguyen C.T., Fraser R.G., Tan R. (2018). Longitudinal changes in retinotopic rod function in intermediate age-related macular degeneration. Invest Ophthalmol Vis Sci.

[40] Hsu S.T., Thompson A.C., Stinnett S.S. (2019). Longitudinal study of visual function in dry age-related macular degeneration at 12 months. Ophthalmol Retina.

[41] Berlin A., Fischer N.A., Clark M.E. (2024). Quantitative autofluorescence at AMD’s beginnings highlights retinal topography and grading system differences: ALSTAR2 baseline. Ophthalmologica.

[42] Welker S.G., Pfau M., Heinemann M. (2018). Retest reliability of mesopic and dark-adapted microperimetry in patients with intermediate age-related macular degeneration and age-matched controls. Invest Ophthalmol Vis Sci.

[43] Csaky K.G., Richman E.A., Ferris F.L. (2008). Report from the NEI/FDA ophthalmic clinical trial design and endpoints symposium. Invest Ophthalmol Vis Sci.

[44] West S.K., Rubin G.S., Broman A.T. (2002). How does visual impairment affect performance on tasks of everyday life?: the SEE Project. Arch Ophthalmol.

[45] Rubin G.S., Bandeen-Roche K., Huang G.H. (2001). The association of multiple visual impairments with self-reported visual disability: SEE project. Invest Ophthalmol Vis Sci.

[46] Carver R.P. (1992). Reading rate: theory, research, and practical implications. J Read.

[47] Rubin G.S. (2013). Measuring reading performance. Vis Res.

[48] Weinreb R.N., Kaufman P.L. (2011). Glaucoma research community and FDA look to the future, II: NEI/FDA Glaucoma Clinical Trial Design and Endpoints Symposium: measures of structural change and visual function. Invest Ophthalmol Vis Sci.

[49] Jackson G.R., Scott I.U., Kim I.K. (2014). Diagnostic sensitivity and specificity of dark adaptometry for detection of age-related macular degeneration. Invest Ophthalmol Vis Sci.

[50] Owsley C., Clark M.E., Huisingh C.E. (2016). Visual function in older eyes in normal macular health: association with incident early age-related macular degeneration 3 Years later. Invest Ophthalmol Vis Sci.

[51] Grewal M.K., Chandra S., Gurudas S. (2022). Functional clinical endpoints and their correlations in eyes with AMD with and without subretinal drusenoid deposits-a pilot study. Eye.

[52] Kumar H., Guymer R.H., Hodgson L.A.B. (2022). Exploring reticular pseudodrusen extent and impact on mesopic visual sensitivity in intermediate age-related macular degeneration. Invest Ophthalmol Vis Sci.

[53] Zhang Y., Sadda S.R., Sarraf D. (2022). Spatial dissociation of subretinal drusenoid deposits and impaired scotopic and mesopic sensitivity in AMD. Invest Ophthalmol Vis Sci.

[54] Flamendorf J., Agrón E., Wong W.T. (2015). Impairments in dark adaptation are associated with age-related macular degeneration severity and reticular pseudodrusen. Ophthalmology.

[55] Lad E.M., Fang V., Tessier M. (2022). Longitudinal evaluation of visual function impairments in early and intermediate age-related macular degeneration patients. Ophthalmol Sci.

[56] Chakravarthy U., Bailey C.C., Scanlon P.H. (2020). Progression from early/intermediate to advanced forms of age-related macular degeneration in a large UK cohort: rates and risk factors. Ophthalmol Retina.

[57] Liu W.S., You J., Ge Y.J. (2023). Association of biological age with health outcomes and its modifiable factors. Aging Cell.

