# Supplementary material for: Heterogeneous Visual Function Deficits in Intermediate Age-Related Macular Degeneration: A MACUSTAR Report
Source: Ophthalmol Sci. 2025 Jan 13;5(4):100708. doi: 10.1016/j.xops.2025.100708 (PMC11985047; doi:10.1016/j.xops.2025.100708)
Supplement: Table S4 [file mmc1.docx]

| **Number of reference limits breached** | **iAMD**  **n (%)** |
| --- | --- |
| 0 | 178 (30.4%) |
| 1 | 157 (26.8%) |
| 2 | 92 (15.7%) |
| 3 | 51 (8.7%) |
| 4 | 37 (6.3%) |
| 5 | 35 (6.0.%) |
| 6 | 19 (3.2%) |
| 7 | 12 (2.1%) |
| 8 | 4 (0.7%) |

Table 4: Summary of iAMD participants breaching 0 – 8 reference limits

Number and proportion of iAMD participants breaching 0 through 8 worse than reference limits.

*AMD: age-related macular degeneration; i: intermediate.*
