# Supplementary material for: Heterogeneous Visual Function Deficits in Intermediate Age-Related Macular Degeneration: A MACUSTAR Report
Source: Ophthalmol Sci. 2025 Jan 13;5(4):100708. doi: 10.1016/j.xops.2025.100708 (PMC11985047; doi:10.1016/j.xops.2025.100708)
Supplement: Table S5 [file mmc2.docx]

| **Visual function measure** | **n (%) with valid data** | **Reference**  **Limit based on worse of V2 and V3** | **n (%) of iAMD participants breaching worse reference limit** |
| --- | --- | --- | --- |
| BCVA | 584 (99.8%) | > 0.11 LogMAR | 120 (20.5%) |
| LLVA | 583 (99.6%) | > 0.32 LogMAR | 141 (24.1%) |
| MAT | 584 (99.8%) | > 0.58 LogMAR | 104 (17.8%) |
| PR-CS | 583 (99.6%) | < 1.45 LogCS | 112 (19.1%) |
| SPS | 548 (93.7%) | < 97 wpm | 63 (10.8%) |
| MesAT | 527 (90.1%) | < 20.7 dB | 73 (12.5%) |
| ScoAT | 496 (84.8%) | < 16.2 dB | 89 (15.2%) |
| RIT | 408 (69.7%) | > 6.48 mins | 160 (27.4%) |

Table 5: Summary of secondary reference limits and proportion of iAMD participants breaching.

Number and proportion iAMD participants breaching secondary worse than reference limits for each visual function test calculated as a proportion of the complete iAMD cohort (585).

*BCVA: best corrected visual acuity; LLVA: low luminance visual acuity; MAT: Moorfields acuity test; PR-CS: Pelli-Robson contrast sensitivity; SPS: Small Print Standardised International Reading Speed Test; MesAT: Mesopic average threshold; ScoAT: Scotopic average threshold; RIT: Rod Intercept Time; LogMAR: logarithm of the minimum angle of resolution; LogCS: logarithm of contrast sensitivity; wpm: words per minute; dB: decibel, mins: minutes.*
