# Supplementary material for: Heterogeneous Visual Function Deficits in Intermediate Age-Related Macular Degeneration: A MACUSTAR Report
Source: Ophthalmol Sci. 2025 Jan 13;5(4):100708. doi: 10.1016/j.xops.2025.100708 (PMC11985047; doi:10.1016/j.xops.2025.100708)
Supplement: Table S6 [file mmc3.docx]

| **Number of reference limits breached** | **iAMD**  **n (%)** |
| --- | --- |
| 0 | 225 (38.5%) |
| 1 | 151 (25.8%) |
| 2 | 84 (14.4%) |
| 3 | 44 (7.5%) |
| 4 | 31 (5.3%) |
| 5 | 27 (4.6%) |
| 6 | 11 (1.8%) |
| 7 | 10 (1.7%) |
| 8 | 2 (0.3%) |

Table 6: Summary of iAMD participants breaching 0 – 8 secondary reference limits

Number and proportion iAMD participants breaching 0 through 8 secondary worse than reference limits.

*AMD: age-related macular degeneration; i: intermediate.*
